# Relationship between the sectional area of the rectus capitis posterior minor and the to be named ligament from 3D MR imaging

**DOI:** 10.1186/s12891-020-3123-x

**Published:** 2020-02-14

**Authors:** Mei-Yu Sun, Xu Han, Meng-Yao Wang, Dian-Xiu Ning, Bin Xu, Li-Zhi Xie, Sheng-Bo Yu, Hong-Jin Sui

**Affiliations:** 1 Department of radiology, the first affiliated hospital of Dalian Medical University, Dalian, China; 2 GE Healthcare, Beijing, China; 3 Department of Anatomy, College of Basic Medicine, Dalian Medical University, Dalian, 116044 People’s Republic of China

**Keywords:** Myodural bridge, To be named ligament, Magnetic resonance imaging, Rectus capitis posterior minor, Sectional area

## Abstract

**Background:**

To evaluate the maximal sectional area (SA) of the rectus capitis posterior minor (RCPmi) muscle and its potential correlation with to be named ligament (TBNL) in the suboccipital area using 3D MR imaging.

**Methods:**

A total of 365 subjects underwent sagittal 3D T_2_WI MR imaging of the RCPmi and TBNL. Among them, 45 subjects were excluded due to a particular clinical history or poor image quality. Finally, 320 subjects met the inclusion criteria, including 138 men and 182 women. The 624 RCPmi muscles were classified into positive and negative groups according to their attachment to the TBNL. Two experienced radiologists manually measured the maximum SA of the RCPmi muscle on the parasagittal image with a 30° deviation from the median sagittal plane. The correlations between the SA and the subject’s age, height, BMI, gender, handedness, and age-related disc degeneration were tested by Spearman analysis. The SA differences between different groups were compared using independent samples *t*-test.

**Results:**

A total of 123 RCPmi-TBNL attachments were identified in the positive group, while 501 RCPmi muscles were identified in the negative group. The SA of the 624 RCPmi muscles was 62.71 ± 28.72 mm^2^ and was poorly correlated with the subject’s age, BMI, or handedness, with no correlation with age-related disc degeneration. A fair correlation was found between the SA and the body height in the whole group, and poor correlation in each male/female group. The SA of the RCPmi muscle in males was significantly bigger than that in women ([75.54 ± 29.17] vs. [52.74 ± 24.07] mm^2^). The SA of RCPmi muscle in the positive group was significantly smaller than that in the negative group ([55.95 ± 26.76] mm^2^ vs. [64.37 ± 28.97] mm^2^).

**Conclusions:**

Our results revealed a significantly smaller SA of the RCPmi in subjects with RCPmi-TBNL attachment. Besides, a larger SA of the RCPmi was correlated with the male gender. These findings suggest that the SA of the RCPmi ought to be interpreted with care for each patient since there could be considerable variations.

## Background

The myodural bridge (MDB) is a connective tissue band that connects the suboccipital muscles and nuchal ligament (NL) with the cervical spinal dura mater (SDM) [1–6]. It has been recently confirmed as a conserved structure in mammals [7–9]. The MDB has a critical role in transmitting tensile force from its muscular and ligamentous components to the SDM, which has an essential role in the etiology of headache and cervicocephalic pain syndrome [10–14]. The rectus capitis posterior minor (RCPmi) was the first suboccipital muscle identified to attach to the dorsal cervical SDM at the posterior atlanto-occipital (PAO) interval [1]. The architecture of muscle, particularly the cross-sectional area (CSA), is a predictor of its force generation [15]. Previous research has focused on the CSA or relative CSA of the RCPmi muscle as biomechanical contributors to cervicocephalic pain syndrome, mild traumatic brain injury (mTBI), and headache syndromes [10–13, 16]. Several studies have shown that the CSA of adult RCPmi muscles is negatively correlated with the severity of chronic headaches [10–13, 16]. Fernfindez-de-las-Penas et al [10, 11] have shown that reduced axial CSA of the RCPmi is correlated with symptom severity in patients with chronic tension-type headache (CTTH) and mTBI. Yet, in contrast to these studies, Yuan et al have reported increased sectional area (SA) of RCPmi muscle in patients with chronic headaches [13]. To correctly assess the effect of the RCPmi muscle’ SA on symptom severity in patients with headache syndrome, it is necessary to thoroughly clarify the magnitude of the variations in the SA of the RCPmi muscles in asymptomatic controls. So far, this issue has not yet been comprehensively addressed. Consequently, an assessment of the SA of the RCPmi muscle in asymptomatic controls is essential for an accurate evaluation of the effect of the SA of the RCPmi muscle in symptomatic patients.

Magnetic resonance imaging (MRI) is a powerful noninvasive imaging tool used to detect the SA of the RCPmi muscle. Though two-dimensional (2D) axial MR images are considered optimal for evaluating the SA of the RCPmi muscle, experimental variables, such as selected image level and variable neck posture, can lead to significant error [17, 18]. Because the reduction in slice thickness lessens partial volume effects that may induce inaccuracy of SA measurement, three-dimensional (3D) MRI may facilitate accurate SA measurement of the RCPmi due to its thinner slices and isotropic post-processed reconstruction. In the present study, physiologic CSA was not calculated though it is more commonly used in conventional calculations to determine muscle atrophy or hypertrophy [12, 16–18]. A narrow pointed tendon attaches the RCPmi to the tubercle on the posterior arch of the atlas. As it ascends, it broadens before attaching to the medial part of the inferior nuchal line and the occipital bone between the inferior nuchal line and the foramen magnum. The physiological CSA of each fan-shaped RCPmi cannot be accurately calculated due to underlying difficulties in taking the identical measurement plane, which could be influenced by factors such as, selected scanning level, atlanto-occipital interspace distance, and neck posture [17, 18]. Referring to the method described by Yuan et al, the angle between the medial and lateral borders of the RCPmi was measured, and the value was (60.7 ± 4.4)°. Hence, 60° from the midline represented the mean of the orientation in the different subjects. Thirty degrees with the largest RCPmi muscle were set as the optimal angle for a better view of the muscle [13]. Therefore, we measured the maximal SA of the RCPmi muscles on the parasagittal image with a 30° deviation from the median sagittal plane through the mid-posterior arch of atlas and the occipital bone. Nevertheless, to the best of our knowledge, no studies are reporting the maximum SA in the RCPmi muscles using 3D MRI, which could be used to determine the variance in SA of the RCPmi muscle for related pathologic studies.

Multiple studies have confirmed the binding of the NL attachment to the cervical SDM via the PAO and posterior atlantoaxial (PAA) intervals [3, 5, 19]. In 2014, Zheng’s study confirmed that the local enhancement of the NL emanated from the posterior border of NL projecting forward and upward to the cervical dura mater, which she termed as the *to be named ligament* (TBNL) [20]. The TBNL may participate in cervicogenic pain syndrome by bridging the dorsal extensor musculature of the cervical spine to the pain-sensitive dura mater. In 2017, Yuan et al found that 11.43% of the RCPmi muscles gave off muscular bundles that merged and terminated at the TBNL whose morphology was influenced by this additional connection [21]. Yet, whether this physical connection from the RCPmi to the TBNL affects the maximum SA of the RCPmi muscle remains unclear.

The SA of RCPmi muscle has been suggested as a potential contributor to headache and cervicogenic pain symptoms; hence, the first aim of this study was to determine the anatomical SA of the RCPmi muscle using 3D T_2_-weighted MR imaging. The RCPmi and TBNL are both critical components of MDB, and their anatomic connection indicates the possibility of a correlation between them. The second aim was to investigate the relationship between the maximum SA of the RCPmi muscles with the RCPmi-TBNL attachment. We hypothesized that the RCPmi and TBNL worked together as an MDB complex to fulfill their crucial function; therefore, the maximum SA of the RCPmi muscle is negatively correlated with the RCPmi-TBNL connection.

## Methods

### Study population

**Fig. 1:**
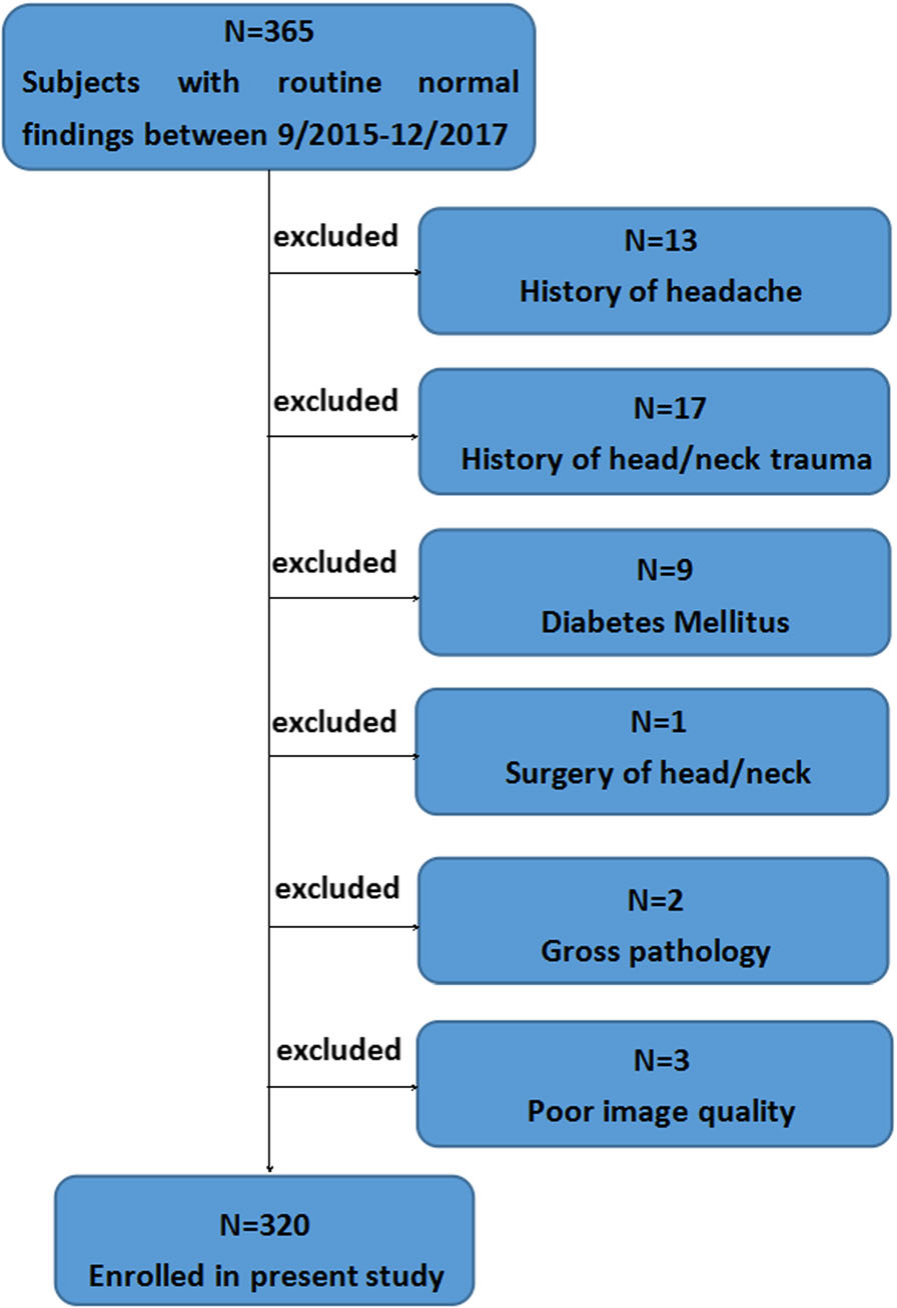
Flow chart demonstrating subjects exclusion criteria

Institutional review board approval was obtained, and written informed consent was waived due to the retrospective nature of the study. A random 365 subjects with routine findings (including normal cervical spine and age-related intervertebral disc degeneration) were enrolled from an ambulatory outpatient population in northern China between September 2015 and December 2017. Forty-two subjects were excluded due to history of headache (*n* = 13), history of head/neck trauma (*n* = 17), diabetes mellitus (*n* = 9), surgery of head and neck (n = 1) and gross pathology (*n* = 2). Another three subjects were excluded due to poor image quality. The flow chart demonstrates the subjects’ exclusion criteria (Fig. 1). Height and body weight were also measured; the body mass index (BMI) was calculated as weight in kilograms (kg) divided by the square of height (m). In addition, the handedness of all participants was recorded.

Three hundred and twenty subjects (mean age 43.40 years, mean height 167.21 cm, 297 right-handed and 23 left-handed) were enrolled, including 138 men (mean age 43.36 years, mean height 174.09 cm) and 182 women (mean age 43.43 years, mean height 161.99 cm). The mean BMI was 23.95 Kg/m^2^ (Table 1).

**Table 1:** Descriptive characteristics and correlation with SA of RCPmi (*n* = 320)

General characteristics	Result	*r*	*P*
Age (year)*	43.40 ± 15.45	− 0.105	0.009
Height (cm)*	167.21 ± 8.48	**0.429**	*<* 0.001
Male	174.09 ± 6.22	0.216	*<* 0.001
Female	161.99 ± 5.82	0.239	*<* 0.001
BMI (Kg/m^2^)*	23.95 ± 3.65	0.161	*<* 0.001
Gender^#^		**0.391**	*<* 0.001
Male	138, (43.13)		
Female	182, (56.87)		
Handedness^#^		0.132	0.001
Right-handed	297, (92.81)		
Left-handed	23, (7.19)		
Age-related disc degeneration^#^		0.005	0.908
None	268, (83.75)		
Yes	52, (16.25)		

*SA* Sectional area, *BMI* Body mass index

* Data are mean· standard deviation, # Data are number (percentage)

### MR imaging

A 3.0 T/1.5 T General Electric (GE) Magnetic Resonance Scanner (Signa, HDxt, GE healthcare, USA) was used to scan the RCPmi muscle and the TBNL of each subject in the study. Subjects were scanned in the supine position in the magnet utilizing a dedicated spinal phased-array coil that covered the whole neck and suboccipital region. The sagittal 3D T_2−_weighted MR imaging was performed with the following parameters: TR: 2500 ms, TE: 70 ms, slice thickness: 1.2/0.6 mm overlap, flip angle: 90°, matrix: 256 × 256, FOV: 24 × 24 cm. The total imaging time was approximately 8 min.

### MR imaging analysis

**Fig. 2:**
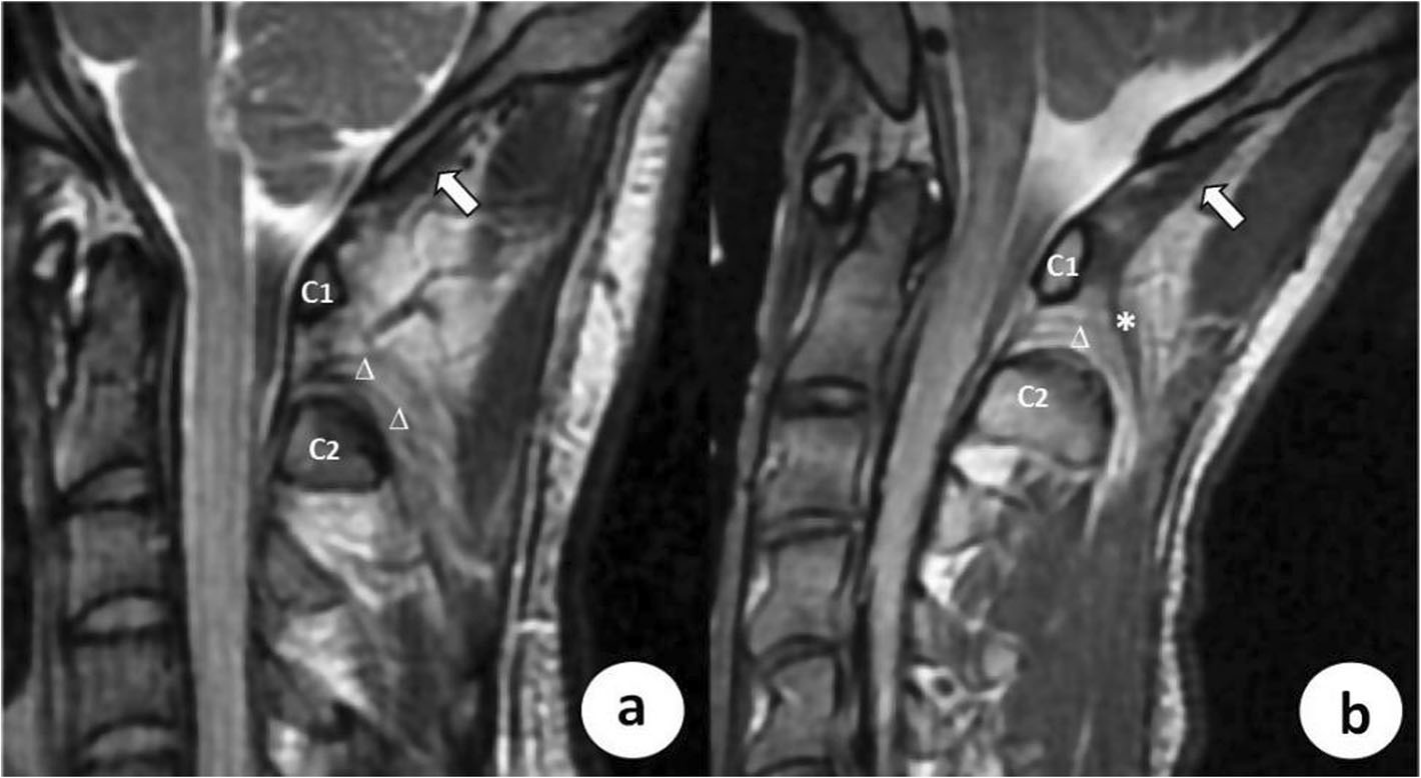
RCPmi muscles with and without an attachment of the RCPmi to the TBNL. **a** Oblique parasagittal T_2_-weighted MR image from a RCPmi in the negative group, which demonstrates no visible attachment from the RCPmi (white arrow) to the TBNL (white ∆). **b** Oblique parasagittal T_2_-weighted MR image from a RCPmi in the positive group which shows continuous hypointense strips/bands (white *) emitting from the RCPmi (white arrow) and traveling inferomedially to the TBNL (white ∆). Note: C1: atlas; C2: axis

The 3D T_2_-weighted MR images were interpreted by two radiologists with 12 and 10 years of experience in musculoskeletal imaging. The parasagittal or oblique sagittal images were generated by image reconstruction to determine whether there was an attachment from the RCPmi to the TBNL (RCPmi-TBNL attachment) or not. The RCPmi-TBNL attachments were identified as visible continuous hypointense strips/bands originating from the posteroinferior aspect of the RCPmi muscle and traveling inferomedially to the TBNL through the hyperintense fat in the suboccipital region. If the RCPmi-TBNL attachment was observed, the RCPmi muscle was placed in the positive group; if no RCPmi-TBNL attachment was identified, it was placed in the negative group (Fig. 2). To minimize bias, the two radiologists discussed and agreed on standard classification criteria, and the final decision was based on consensus.

**Fig. 3:**
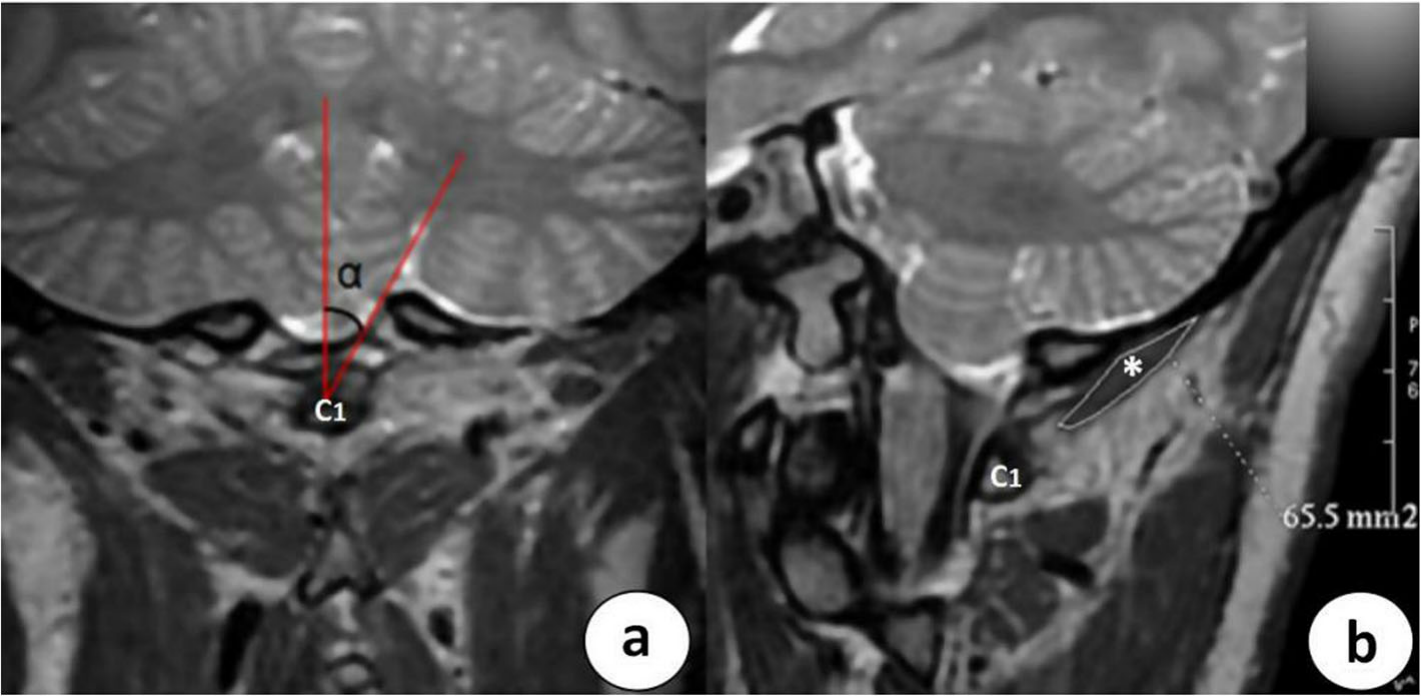
The measurement method for the maximum SA of the RCPmi muscle. **a** Coronal T_2_-weighted MR image across the posterior arch of the atlas. The SA of the RCPmi muscle is measured on an oblique parasagittal slice with a 30° deviation from the mid-posterior arch of the atlas. **b** On the oblique sagittal T_2_-weighted MR image defined in a., the SA of the left RCPmi muscle is given by the delineated area (white*) and has a calculated value of 65.5 mm^2^. Note: SA, sectional area; C1: atlas; α = 30°

Measurements of the maximum SA of the left and right RCPmi muscles were performed by two radiologists with 5 and 4 years of experience in musculoskeletal imaging using Functool software installed on an Advantage Workstation 4.4 (Sun Microsystems, Santa Clara, Calif, USA). These two radiologists were blinded to the name and details of the patients whose images they were analyzing. Besides, the interobserver agreement was systematically assessed. The maximum SA of the RCPmi muscle was measured on the parasagittal image located between the mid-posterior arch of the atlas and the occipital bone with a 30° deviation from the median sagittal plane [13]. Anatomic maximum SA was calculated inside the boundary manually placed around the perimeter of the RCPmi muscle belly (Fig. 3).

### Statistical methods

All statistical analyses were performed using the SPSS 22.0 software (SPSS Inc., Chicago, IL, USA). Data were reported as mean ± SD. The interobserver agreement of the SA measurement was evaluated using the repeated measures ANOVA and intraclass correlation coefficient (ICC). An ICC value < 0.5, between 0.5 and 0.75, between 0.75 and 0.9, and > 0.90 were indicative of poor, moderate, good, and excellent reliability, respectively [22]. The correlations between the SA and the subject’s age, height, BMI, gender, handedness and disc degeneration were tested by Spearman analysis. A Spearman correlation coefficient (r) less than 0.1, between 0.1 and 0.3, between 0.3 and 0.6 was considered negligible, poor and fair correlation, respectively [23]. The gender differences in the positive and negative groups were tested with a chi-squared test. The height differences between male/female and positive/negative groups were compared using an independent samples *t*-test. The SA differences, between the positive and negative groups, male versus female groups, and left versus right sides, were compared using independent samples *t*-test. For all statistical results, a *p*-value < 0.05 was considered statistically significant.

## Results

The descriptive characteristics of 320 subjects are listed in Table 1. The body height in the male group was significantly bigger than that in the female group (*t* = 17.89, *p* < 0.001). Six hundred and twenty-four RCPmi muscles were observed in 320 subjects. The RCPmi-TBNL attachment was identified in 123 RCPmi muscles with a left side to right side ratio of 61:62 and a male to female ratio of 50:73. Moreover, five hundred and one RCPmi muscles were placed into the negative group with a left to right side ratio of 250:251 and male to female ratio of 223:278. There was no statistically significant difference in gender between these two groups (*χ*^2^ = 0.598, *p* = 0.439). There was no statistically significant difference in body height between negative and positive groups [(167.45 ± 8.41) cm vs. (167.09 ± 8.50) cm, *t* = 0.418, *p* = 0.676].

**Fig. 4:**
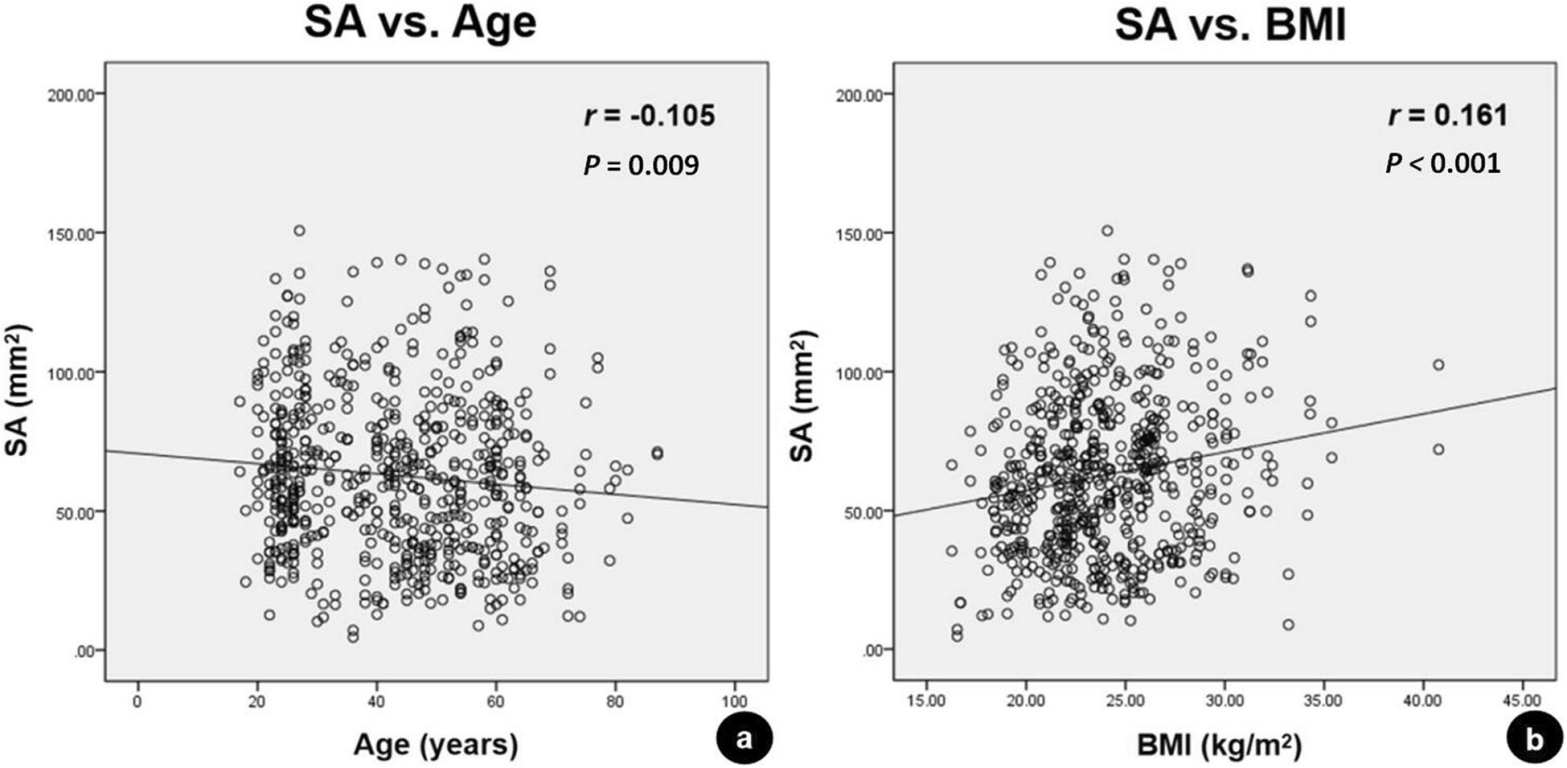
Scatterplots displaying the correlations between the SA and the subject’s age or BMI. Significant linear fittings are marked with solid lines for the SA. The mean SA of the 624 RCPmi muscles was poorly correlated with the subject’s age (**a**) and BMI (**b**). Note: SA, sectional area; BMI, body mass index

**Fig. 5:**
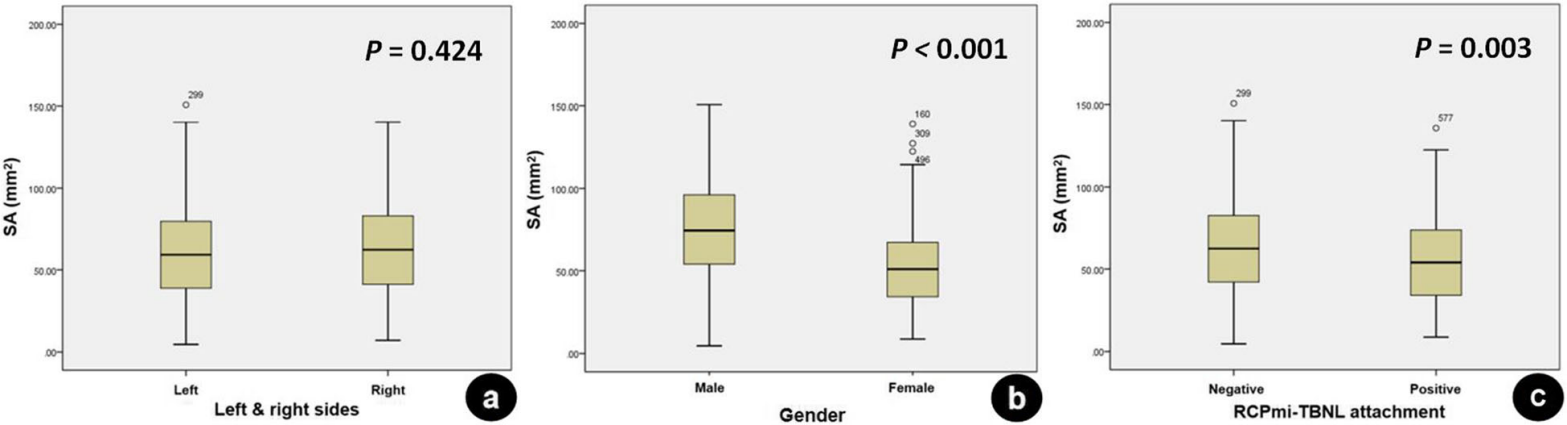
Boxplots showing the mean SA of the RCPmi muscles in left vs. right sides, male vs. female groups, negative and positive RCPmi-TBNL attachment groups. **a** There was no statistically significant difference between the left and right RCPmi muscles. **b** The mean SA of the RCPmi muscles in men was significantly larger than in women. **c** The mean SA of the RCPmi muscles in the positive group was significantly smaller compared to the negative group. Note: SA, sectional area

The ICC of inter-observer agreement of the MRI measurements for the SA of the RCPmi muscles was 0.891 (95%confidence interval 0.886–0.916), which suggested a good reliability. The mean SA was calculated for subsequent analysis. The mean SA of the 624 RCPmi muscles was 62.71 ± 28.72 mm^2^ and was poorly correlated with the subject’s age, BMI, or handedness, with no correlation with age-related disc degeneration (Fig. 4). Yet, SA and gender were fairly correlated. A fair correlation was also found between the SA and the body height in the whole group, and poor correlation in each male/female group (Table 1). The mean SA of the RCPmi muscles in men was significantly larger than in women. There was no statistically significant difference between the left and right RCPmi muscles. The mean SA of the RCPmi muscles in the positive group was significantly smaller compared to the negative group (Table 2, Fig. 5).

**Table 2:** The mean SA (mm^2^) of the RCPmi muscle between different groups

	Sides (n,%)		Gender (n,%)		RCPmi-TBNL attachment (n,%)	
	Left (311,49.84)	Right (313,50.16)	Male (273,43.75)	Female (351,56.25)	Positive (123,19.71)	Negative (501,80.29)
SA	61.79 ± 28.76	63.63 ± 28.70	75.54 ± 29.17	52.74 ± 24.07	55.95 ± 26.76	64.37 ± 28.97
*t*	−0.799		10.443		2.932	
*P*	0.424		**< 0.001**		**0.003**	

All values are presented as mean ± standard deviation

## Discussion

In this study, we determined the mean and variance of the anatomic maximum SA data of the RCPmi muscles in asymptomatic subjects. Furthermore, the obtained results based on non-invasive measurements of SA on 3D T_2_-weighted MR imaging, suggested that a smaller SA of RCPmi muscle might be present in subjects with an RCPmi-TBNL attachment.

The RCPmi was the first deep suboccipital muscle found to be linked to the cervical spinal dura via the myodural bridge. However, its functional role through the MDB is still not fully elucidated [2]. Several reports have shown that changes in the SA of the RCPmi muscle are correlated with the severity of cervicocephalic pain syndrome, mTBI and headache syndrome [10–14]. Accordingly, it is crucial to accurately document the SA of the RCPmi to determine its influence. In the present study, the mean maximum SA of the 624 sampled RCPmi muscles was 62.71 mm^2^. There was a poor correlation with the subject’s age, handedness, or BMI. In addition, a fair correlation was noticed between the SA and the body height in the whole group, and poor correlation in each male/female group. This suggested that gender was an important factor affecting SA of RCPmi muscles. Moreover, gender did account for some of the observed differences between the SA of the RCPmi muscle. The mean SA of the RCPmi muscle in males was significantly bigger than in females. These results were also corroborated by Yuan et al who found that the mean SA of the RCPmi muscle was gender-related [13]. In their study, they scanned subjects with 2D T_2_-weighted MR imaging and used a measurement method similar to ours. Results of SA of the RCPmi were somewhat smaller in our study; however, the results of the study above were based on findings from 2D T_2_-weighted images. The choice of MRI sequence and other scan parameters may also have contributed to this difference, thus making it difficult to quantitatively compare our results with previous studies. The present study is the first to measure the mean and variance of the maximum SA of the RCPmi muscle using 3D T_2_-weighted MR imaging. Knowing of the maximum SA of the RCPmi muscle is important for predicting the forces that it generates and then transmits to the spinal dura via the myodural bridge, which might be crucial for generating certain chronic pain and headache syndromes.

The RCPmi-TBNL attachment is composed of muscular bundles of the RCPmi joining with the TBNL. It has the appearance of a combination structure derived from these two critical myodural components, yet the physiology of this physical connection still remains unknown. Interestingly, the mean SA of the RCPmi in the positive RCPmi-TBNL attachment group was significantly smaller compared to the negative group, which has not been previously described. Furthermore, 19.71% of the RCPmi muscles were attached to the TBNL in the present study. It demonstrated that the physical RCPmi-TBNL connection was more prevalent than it was observed by Yuan et al in 35 adult head-neck specimens [21]. This rather common variant in about 1/5 subjects should be taken into account with the future measuring of the SA of the RCPmi muscle using MR imaging. It is also essential to consider these differences in prospective studies looking at how the SA of the RCPmi muscles relate to head and neck pain syndromes. Both the RCPmi muscle and the TBNL are important components that connect to the dorsal cervical spinal dura via the MDB. The difference we observed in the SA of the RCPmi muscle with and without additional attachment to the TBNL may suggest a similar functional role for the RCPmi muscle and the TBNL. Assuming that the RCPmi and the TBNL are one complex structure, the existence of RCPmi-TBNL attachment would be reflected in a comparatively smaller SA of the RCPmi muscle. With more physical connections observed between myodural bridge and relevant musculature/ligament, we can get more evidence about the myodural bridge complex [24]. This may indicate that the RCPmi muscle and the TBNL work together as a MDB complex, which helps to understand better the potential role they have in the pathogenesis of cervicogenic pain syndromes [10–13]. Identifying the variations in the RCPmi muscle morphometry across a specific asymptomatic population provides the basis needed for future studies to assess the potential relationship of the SA to headache syndromes and chronic cervical pain.

The present study has few limitations. We did not account for other influential factors that might affect the SA of the RCPmi muscle, including the level of physical activity and workload. Yet, as the RCPmi muscle is small and its primary function does not involve carrying the load and producing large torques, it should not be affected by overall physical activity levels. The most common influential factors, including gender, age, left- or right-handed, left- or right-side, height and BMI, were assessed in this study, but further research should try to determine other possible factors, such as atlanto-occipital distance. Furthermore, logistic regression and modeling should be applied in future studies. For those patients with anatomical variability in fibre/muscle orientation or even absence of the RCPmi muscle, a new assessment method may be required. Besides, further studies are needed to investigate the contribution of other components of the MDB, including the rectus capitis posterior major and the obliquus capitis inferior muscles, to the clinical manifestation of cervicogenic headaches. Future work concerning the function of the MDB should also be expanded to include data from other cervical deep extensor muscles attached to nuchal ligament.

## Conclusions

The current study looked at the SA of the RCPmi muscle using 3D T_2_-weighted MR imaging. Our results found a significantly smaller SA of the RCPmi in subjects with an RCPmi-TBNL attachment, suggesting a similar functional role for the RCPmi and the TBNL as part of the MDB. Besides, a larger SA of the RCPmi was correlated with the male gender. These findings suggest that the SA of the RCPmi ought to be interpreted with care for each patient since considerable variations might occur. The present study may serve as a baseline for further investigation of the RCPmi muscle and TBNL and their clinical significance in cervicogenic pain syndromes.

## Data Availability

The datasets used and/or analyzed during the current study are available from the corresponding author on reasonable request.

## Abbreviations

2D: Two-dimensional
3D: Three-dimensional
BMI: Body mass index
CSA: Cross-sectional area
CTTH: Chronic tension-type headache
MDB: Myodural bridge
MRI: Magnetic resonance imaging
mTBI: mild traumatic brain injury
NL: Nuchal ligament
PAA: Posterior atlantoaxial
PAO: Posterior atlanto-occipital
RCPmi: Rectus capitis posterior minor
SA: Sectional area
SDM: Spinal dura mater
TBNL: To be named ligament
